# Splenic hamartoma with bizarre stromal cells: a case report and literature review

**DOI:** 10.1186/s13000-018-0687-y

**Published:** 2018-01-22

**Authors:** Na Cheng, Jianning Chen, Yuhang Pan, Ye Jiang, Jing Zhou, Chunkui Shao

**Affiliations:** Department of Pathology, The Third Affiliated Hospital, Sun Yat-sen University, 600 Tianhe Rd, Guangzhou, 510630 China

**Keywords:** Spleen, Splenic hamartoma, Bizarre stromal cells, Immunohistochemistry

## Abstract

**Background:**

Splenic hamartoma is a rare benign vascular proliferative lesion composed of unorganized sinusoid-like channels lined with plump or flat endothelial cells and characterized by a CD8-positive immunophenotype of the lining cells. Scattered bizarre stromal cells can be found in some splenic hamartomas. The presence of splenic hamartoma with bizarre stromal cells is extremely rare and these bizarre cells make it possible to be regarded as a malignancy. Recognition of this rare histologic variant will help to avoid diagnostic confusion and overtreatment of this benign entity.

**Case presentation:**

We report a case of a 40-year-old man with occasional left-sided waist back pain. A splenic space-occupying lesion was detected by ultrasound and magnetic resonance imaging. Microscopically bizarre large cells were scattered throughout the splenic hamartoma. The cells exhibited atypical nuclei, scarcely visible cytoplasm, and vesicular chromatin, and they did not form expansile clusters and lacked mitotic activity. An immunohistochemical panel was performed. The bizarre cells strongly expressed vimentin, and the Ki-67 index was very low. The lesion was diagnosed as a splenic hamartoma with bizarre stromal cells.

**Conclusions:**

To the best of our knowledge, this is the first systematic review on a splenic hamartoma with bizarre stromal cells; only six cases have been described in the literature. Proper identification is important to secure adequate treatment.

## Background

Splenic hamartomas, originally described by Rokitansky in 1861, have been documented in fewer than 200 cases to date in the literature. Splenic hamartoma is a very rare benign vascular lesion and has also been called a splenoma or spleen within a spleen. They are usually asymptomatic and discovered incidentally, particularly in the adult [1]. Splenic hamartoma consists of disorganized sinusoid-like channels such as red pulp tissue, the lining cells of which can be highlighted by CD8 immunopositivity, while no white pulp elements are observed in the lesion [2–4].

When some large and atypical stromal cells appear in splenic harmartoma, it can be challenging to determine whether the hamartoma represents a benign or malignant lesion. Cheuk et al. first reported splenic hamartoma with bizarre stromal cells in 2005 [5]. To date, only six such cases have been described in the literature. These stromal cells with morphological diversity cause difficulties in diagnosis and raise a mimicker of malignancy. Presented here is an asymptomatic patient with a round and solid lesion in the spleen. We present our observations about morphological features and the immunohistochemical data of bizarre stromal cells in this splenic hamartoma.

## Case presentation

### Clinical features

**Fig. 1:**
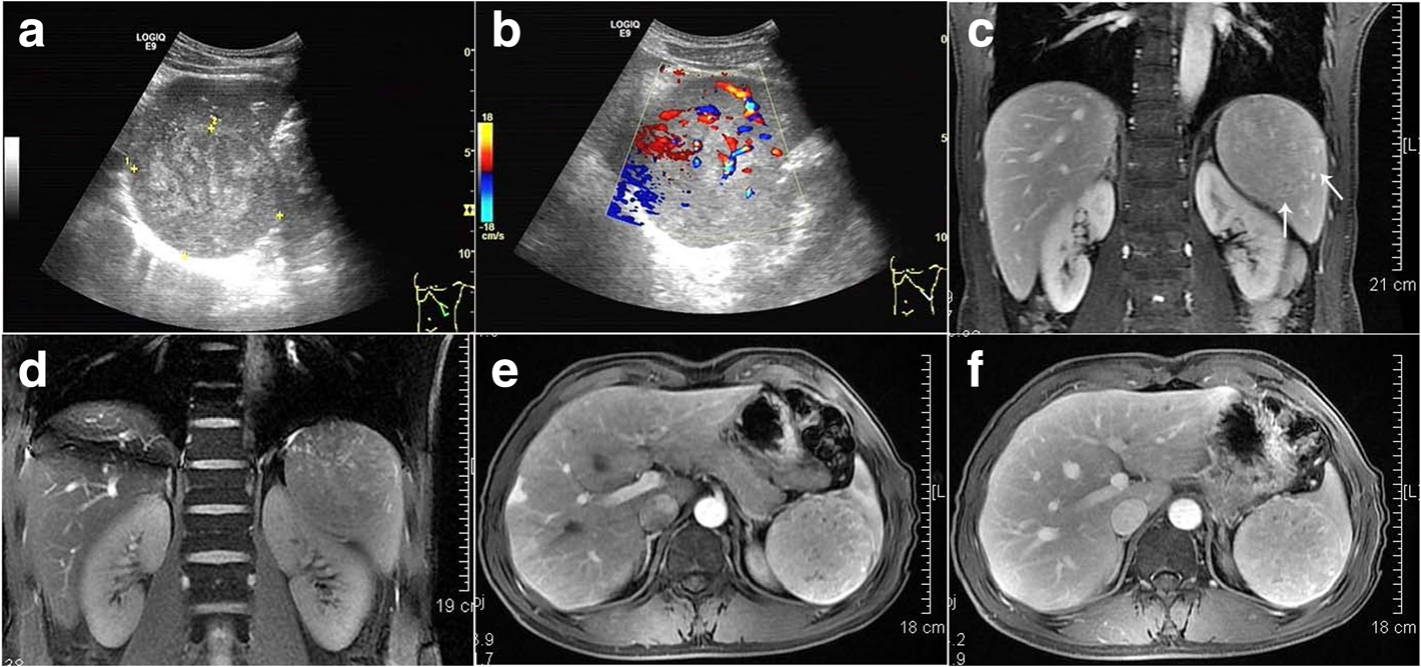
A splenic mass was found incidentally in a 40-year-old man. The abdominal ultrasound image (**a**) showed a round mixed echogenicity mass in the spleen, in which inhomogeneous echogenicity with patchy hyperechoic and iso-echoic foci were noted. Internal color Doppler flow was also observed (**b**). Magnetic resonance imaging (MRI) displayed a nearly iso-signal lesion (arrow) compared to the splenic parenchyma both on T1-weighted (**c**) and T2-weighted (**d**) images. The mass demonstrated relatively homogenous enhancement to the same degree as that of the splenic parenchyma on arterial phase (**e**) and delayed phase (**f**)

A 40-year-old man with no significant medical history, had experienced occasional left-sided waist back pain for 11 months. No organomegaly was noted in the waist on physical examination. Abdominal ultrasound demonstrated a round mixed echogenicity mass in the spleen, with internal color Doppler flow (Fig. 1a, b). Within the mass, the inhomogeneous echogenicity with patchy hyperechoic and iso-echoic foci were noted. Magnetic resonance imaging (MRI) displayed a hyper-enhancing abnormal signal lesion on T1-weighted and T2-weighted images (Fig. 1c–f), the result of which was considered an inflammatory myofibroblastic tumor (IMT). The differential diagnosis included an atypical hemangioma and lymphoproliferative disorders. No sign of infiltration of adjacent organs was found but malignancy could not be ruled out. The patient underwent splenectomy and has remained well in follow-up for 3 years.

### Pathologic findings

**Fig. 2:**
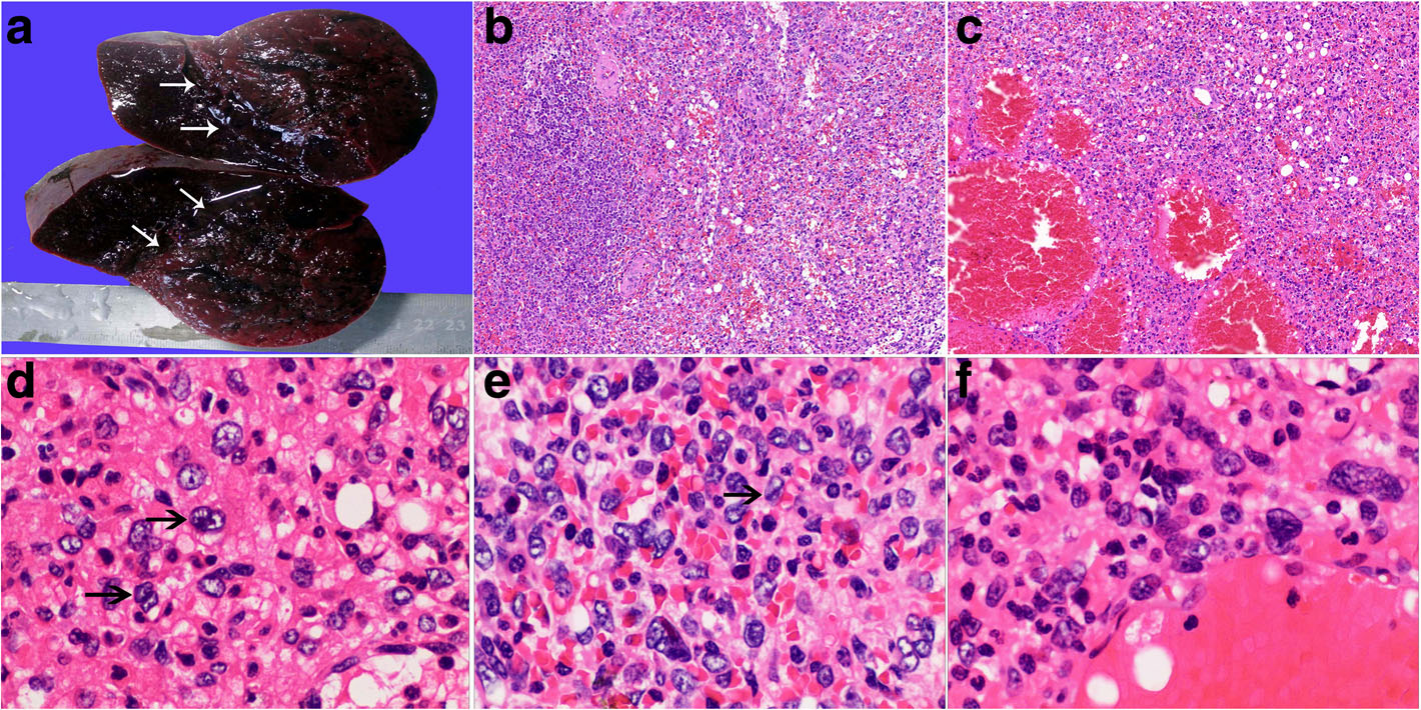
**a** The cut surface of the resected spleen, illustrating a solitary, round and dark-red mass with expansile growth compressing the surrounding parenchyma (10% buffered formalin-fixed). **b** Low-power view showing the lesion (right field) composed of unorganized sinusoid-like channels without malpighian corpuscles, less well demarcated from the adjacent normal splenic tissue (left field) (H&E, × 100). **c** In some areas, the cavernous vascular channels were filled with erythrocytes and foci of fat vacuoles can be noted (H&E, × 100). **d-f** Many bizarre large cells are scattered in the stroma throughout the lesion, with oval, reniform, multilobulated, or convoluted nuclei. The chromatin is pale, granular or vesicular. Sometimes nuclear grooves (e, arrow) are present. Note large cells (d, arrows) with double nuclei and apparent eosinophilic nucleoli mimicking Reed-Sternberg cells in classical Hodgkin’s lymphoma. (H&E, × 400)

Macroscopically, the resected spleen measured 13 cm × 7.8 cm × 6.5 cm. Cut sections of the spleen revealed a solitary, well-circumscribed but unencapsulated, round and solid mass with dark red color sized 7.5 cm × 7 cm × 6.5 cm. The lesion demonstrated expansile growth compressing the adjacent normal splenic parenchyma (Fig. 2a). A small accessory spleen (approximately 0.6 cm in diameter) was also noted.

On histologic examination, the lesion was less well demarcated from the adjoining parenchyma and compressed the surrounding tissue (Fig. 2b). It was composed of unorganized slit-like, narrow tubular or cavernous vascular channels lined with plump or flat endothelial cells. These vascular channels contained erythrocytes, and the extravasation of red cells was also prominent (Fig. 2c). Variable numbers of small lymphocytes, neutrophils, plasma cells, and siderophages infiltrated the loose stroma. There were scattered small aggregates of lymphocytes while no organized lymphoid follicles of the white pulp were observed. Foci of fat vacuoles were noted, and there was no extramedullary hematopoiesis (Fig. 2c).

The most striking population was isolated bizarre large cells that were scattered throughout the lesion. These bizarre stroma cells did not line or appear within the vascular lumens, nor were they aggregated around the sinusoid-like channels (Fig. 2d–f). They exhibited irregular morphology of nuclei, such as oval, reniform, multilobulated, convoluted, or deeply cleaved with scarcely visible cytoplasms. The chromatin was finely granular or vesicular. Intranuclear pseudoinclusions and nuclear grooves occasionally were noted. Nucleoli were inconspicuous and sometimes prominent in some bizarre cells. Mitotic activity, necrosis, or infiltrative growth pattern was not identified.

The rest of the spleen and the accessory spleen presented normal components of red and white pulp.

### Immunohistochemical findings

**Fig. 3:**
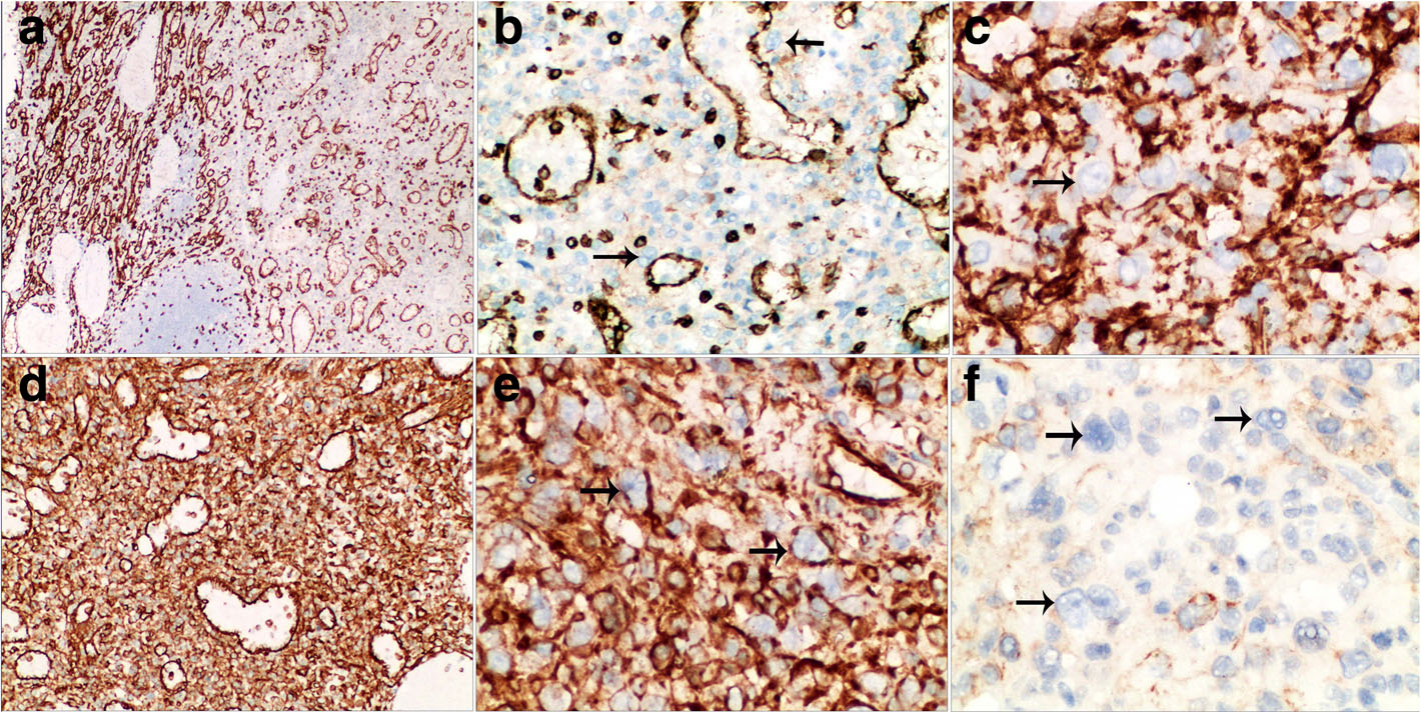
**a-b** Immunostaining for CD8.The sinusoid-like structures are highlighted by CD8 in the splenic hamartoma, in addition to some small lymphocytes (a, × 40; b, × 200). Normal red pulp is shown for comparison (left field) (a, × 40). **c** Immunostaining for CD31 highlights the lining cells of disorganized sinusoid-like channels and histiocytes (c, × 400). **d**-**e** Immunostaining of vimentin is diffusely positive in the lesion (d, × 200; e, × 400). **f** Immunostaining of SMA is negative (× 400). For all markers, the bizarre stromal cells (arrows, b, c, f) are negative, except for vimentin (arrows, e)

An immunohistochemical panel (Table 1) was performed. On immunohistochemical staining, the lining cells of sinusoid-like channels stained positively for CD8, consistent with the endothelia of normal splenic sinuses (Fig. 3a, b). CD31 and Fli-1 were also positive for lining cells while CD34 was focal positive (Fig. 3c). Histiocytes were revealed by CD68 and CD31. Immunostain for vimentin was diffusely positive (Fig. 3d, e).

**Table 1:** Antibodies used for immunohistochemical staining

Antibody	Major Specificity	Source	Dilution
Epithelial marker			
Cytokeratin (AE1/AE3)	Pan-cytokeratin	Novocastra	1:100
Mesenchymally-derived cell marker			
Vimentin (V9)	Mesenchymally-derived cell	Dako	1:1000
Endothelial markers			
CD31 (JC/70A)	Endothelial cells, megakaryocytes, plasma cells	Dako	1: 50
CD34 (QBEnd/10)	Endothelial cells, some stromal cells	Dako	1:200
Fli-1 (G146-222)	Endothelial cells, Ewing’s sarcoma	Maixin	RTU
Lymphoid and myeloid markers			
CD3 (LN10)	T cells	Novocastra	1:100
CD20 (L26)	B cells	Novocastra	1:250
CD8 (1A5)	Cytotoxic T cells, splenic sinus-lining cells	Novocastra	1: 50
CD15 (Carb-3)	Reed-Sternberg cells, granulocytes, histiocytes	Dako	RTU
CD30 (Ber-H2)	Activated lymphoid cells, Reed-Sternberg cells, anaplastic large cell lymphoma	Dako	1:40
ALK (5A4)	Anaplastic large cell lymphoma, HL	Novocastra	RTU
Pax-5 (SP34)	B cells	Ventana	RTU
CD61 (2f2)	Megakaryocyte	Novocastra	1:100
MPO (59A5)	Myeloid cells	Novocastra	1:1000
Histiocyte and monocyte marker			
CD68 (514H12)	Histiocytes and monocytes	Novocastra	1:100
Follicular dendritic cell markers			
CD21 (2G9)	Follicular dendritic cells	Novocastra	RTU
CD35 (RLB25)	Follicular dendritic cells	Novocastra	1:50
Myogenic markers			
Smooth muscle actin (αsm-1)	Muscle cells, myofibroblasts, pericytes	Novocastra	1:200
Desmin (DE-R-11)	Muscle cells, myofibroblasts	Novocastra	1:200
Langerhans cell markers			
S-100 protein (antiserum)	Melanocytes, dendritic cells, nerve sheath cells, cartilage cells myoepithelial cells, fat cells	Dako	1:1000
CD1α (010)	T cells, Langerhans cells, thymocyte	Dako	1:50
Proliferation-related marker			
Ki-67 (MIB-1)	Cell proliferation activity	Dako	1:100

*Abbreviations: RTU* ready-to-use

The bizarre stroma cells were negative for all other markers except for vimentin.

## Discussion

Although vascular neoplasm is the most frequent primary neoplasm of the spleen, splenic hamartoma is a rare benign “tumor” (about three cases in 200,000 splenectomies) occurring in any age group, with no gender predilection and usually without symptoms [1, 6]. Splenic hamartoma is generally found incidentally during imaging tests performed for other reasons or at autopsy. Clinical manifestations associated with larger masses are more often encountered among women, suggesting a hormonal influence. In addition, symptoms are reported most frequently in pediatric patients. Although the popularity of modern radiologic imaging techniques, such as ultrasound, color Doppler ultrasound, computed tomography (CT), and MRI, make it possible for early detection of the splenic hamartoma, definitive diagnosis depends on tissue examination.

Splenic hamartoma most commonly is a solitary or multiple, round, well-circumscribed, unencapsulated, and dark red nodule compressing the adjacent normal parenchyma. The lesions vary in size ranging from a few millimeters to 20 cm maximum. Histologically, splenic hamartoma consists of unorganized sinusoid-like channels without interspersed white pulp. The lining cells of sinusoid-like channels are CD8-positive, which is a key immunohistochemical feature for splenic hamartoma [7]. The cells are also positive for CD31, factor VIII–related antigen, and vimentin, while the expression of CD34 is inconsistent in different reports [3, 5, 8–10]. In our case, CD34 shows a focal positive for lining cells. Additionally, T lymphocytes (CD3 +), B lymphocytes (CD20 +), macrophages (CD68 +), and fibroblasts in the loose stroma can be stained by related immunohistochemical markers [2].

The pathogenesis of splenic hamartoma is controversial. Some consider hamartoma as the congenital malformation of the red pulp, excessive and disorganized growth of abnormally formed red pulp, a neoplasm, or a reactive lesion to prior trauma [4, 8, 11]. Some report that splenic hamartoma is associated with other hamartomatous lesions such as tuberous sclerosis [12–14].

Recently, several cases of splenic hamartoma with bizarre stromal cells have been reported as a difficult-to-diagnose variant. To the best of our knowledge, seven cases, including our patient, have been documented to date (Table 2) [5, 15–17]. The patients were 5 women and 2 men, ranging in age from 35 to 64 years (mean, 50.4 years; median, 50 years). None of the patients had evidence of recurrent disease after splenectomy. Microscopically, the large bizarre cells were distributed randomly throughout the stroma of the lesion without association with vascular lumen. These cells with morphological diversity make it possible to misdiagnose this rare benign variant as a malignancy.

**Table 2:** Clinicopathologic features of reported cases of splenic hamartomas with bizarre stromal cells

Source	Sex/Age	Spleen Weight (g)	Size (cm) of Lesions	Gross description	Clinical symptoms	Follow-up
Cheuk [5]	F/63	100	3.7	Well circumscribed, dark red lesion	Incidental radiologic finding of splenic mass during investigation of fever (probably due to urinary tract infection)	NED, 1 year
Cheuk [5]	M/48	171	9	Well circumscribed	Abdominal pain	Well after surgery
Cheuk [5]	F/50	320	7	Well-delineated,soft and red black lesion	Abdominal pain	NED, 1 year
Laskin [15]	F/53	142	4.2* 2.8*2.5	Well-circumscribed, nonencapsulated, homogeneous tan-red mass	Abdominal pain	Benign clinical course
Yigit [16]	F/35	NM	24.5 * 13.6 *13	Well-circumscribed, dark red colored, soft and solid mass	Chest pain and pelvic pressure, pancytopenia	Well after surgery
Collins [17]	F/64	NM	3.2	bulging, round and dark red mass	History of low-grade malignant fibrous histiocytoma	Benign clinical course
Current case	M/40	–	7.5*7*6.5	Well-circumscribed, unencapsulated, round dark red colored and solid mass	Occasional left-sided waist back pain	Well after surgery

*Abbreviations: NED* no evidence of disease, *NM* not mentioned

For this case, the combined morphologic and immunohistochemical profile supported a diagnosis of splenic hamartoma with bizarre stromal cells that was a benign lesion. Differential diagnosis should be considered, including a group of primary or secondary lesions of the spleen presenting a pattern of spindled cells mixed with bizarre large cells and different kinds of inflammatory cells in the loose stroma. In our case, the most likely differential diagnostic considerations include IMT, follicular dendritic cell (FDC) sarcoma, angiosarcoma, and Hodgkin’s lymphoma.

Inflammatory myofibroblastic tumor is an uncommon neoplasm originally denoted as inflammatory pseudotumor [18]. IMT is a distinctive lesion composed of myofibroblastic spindle cells mixed with an inflammatory infiltration of lymphocytes, plasma cells, and eosinophils. Three basic histological patterns form in the tumor: an edematous myxoid vascular pattern resembling nodular fasciitis; a compact fascicular spindle cell pattern with variable myxoid and collagenized regions; and hypocellular plate-like collagenized pattern resembling a scar or desmoid-type fibromatosis. Immunocytochemistry shows positive staining for vimentin, SMA, desmin, ALK (50% +), and negative staining for S-100 and CD30 [19, 20]. Unlike the splenic hamartoma, myofibroblastic spindle cells of IMT are negative for CD8 and CD31.

FDC sarcoma is a neoplastic proliferation of spindled to ovoid cells dispersed within a prominent lymphoplasmacytic infiltration [21]. Typically, spindled cells show indistinct cell borders and vesicular nuclei. Some are bland-looking while others are enlarged or overtly atypical. FDC sarcoma is positive for one or more of the follicular dendritic markers, such as CD21, CD23, and CD35, and the EBER is tested by in situ hybridization [22]. However, bizarre stromal cells of splenic hamartoma are negative for all the follicular dendritic markers.

Primary splenic angiosarcoma is an extremely rare nonlymphoid malignant neoplasm that originates from the splenic sinusoidal vascular endothelium [23]. The tumor consists of irregular and anastomosing vascular channels lined by atypical endothelial cells with high nuclear grade displaying mitotic activity. Although CD8 staining of the lining cells in splenic angiosarcoma has been reported, the main endothelial cell markers, including CD31, CD34, factor VIII–related antigen, and the histiocytic marker CD68, should exhibit strong positivity [10]. Angiosarcoma has a high rate of metastasis and poor prognosis [24].

Sometimes large stromal cells with double nuclei and apparent eosinophilic nucleoli in splenic hamartoma mimic Reed-Sternberg (R-S) cells in classical Hodgkin’s lymphoma (CHL). CHL is a monoclonal lymphoid neoplasm composed of mononuclear Hodgkin cells and multinucleated R-S cells residing in an abundant admixture of infiltrative non-neoplastic inflammatory cells, histocytes, and fibroblasts [25]. R-S cells are positive for CD30 in nearly all cases and for CD15 in the majority (75-85%) of cases; they are usually negative for CD45 and CD68.

Similarly, the histogenesis of bizarre stromal cells in splenic hamartoma is still elusive, since they demonstrate no specific differentiation to epithelial, endothelial, lymphoid, histiocytic, myeloid, or melanocytic cells. For the reported six cases, the numerous markers tested are negative except a focal and faint positive for desmin in three cases, very focal and equivocal staining for SMA in one case, and a positive for keratin (CAM5.2) and CD30 in another case. According to the immunohistochemical expression, Cheuk et al. and other literature consider that these cells, which may be related to the stromal myoid cells or so-called fibroblastic reticulum cell that are usually present in the red pulp, the periarterial lymphoid sheath, and marginal zone of the spleen, represent a degenerative change accompanied by partial or complete loss of the myoid immunophenotype or undergo immunophenotypic modulation in response to physiological or pathological stimuli [5, 15]. Although bizarre stromal cells in our case failed to react with the above markers, we suspect that they are still a degenerative change. No matter what the immunohistochemical markers express, splenic hamartoma with bizarre stromal cells has benign clinical behavior because the bizarre cells do not form expansile clusters, they lack mitotic activity, and the Ki-67 index is very low.

## Conclusion

Splenic hamartoma is a rare benign vascular proliferative lesion characterized by the CD8-positive immunophenotype of the lining endothelial cells. Bizarre stromal cells without immunohistochemical differentiation can be present in some splenic hamartomas. Awareness of this histologic variant can prevent misdiagnosis of this rare benign entity as a malignant tumor.

## Abbreviations

CHL: Classical Hodgkin lymphoma
CK: Cytokeratin
CT: Computed tomography
FDC: Follicular dendritic cell
IMT: Inflammatory myofibroblastic tumor
MRI: Magnetic resonance imaging
NED: No evidence of disease
NM: Not mentioned
R-S cells: Reed-Sternberg cells
RTU: Ready-to-use
SMA: Smooth muscle actin (αsm-1)
